# The Insider: Impact of the Gut Microbiota on Cancer Immunity and Response to Therapies in Multiple Myeloma

**DOI:** 10.3389/fimmu.2022.845422

**Published:** 2022-03-17

**Authors:** Arianna Brevi, Laura Lucia Cogrossi, Marco Lorenzoni, Benedetta Mattorre, Matteo Bellone

**Affiliations:** ^1^ Cellular Immunology Unit, Department of Immunology, Transplantation and Infectious Diseases, Istituto di Ricovero e Cura a Carattere Scientifico (IRCCS) Ospedale San Raffaele, Milan, Italy; ^2^ Università Vita-Salute San Raffaele, Milan, Italy

**Keywords:** microbiota, multiple myeloma, monoclonal gammopathy of undetermined significance, smoldering multiple myeloma, prevotella, T helper 17, interleukin 17, gut micobiome

## Abstract

The human microbiota is a unique set of microorganisms colonizing the human body and evolving within it from the very beginning. Acting as an insider, the microbiota provides nutrients, and mutualistically interacts with the host’s immune system, thus contributing to the generation of barriers against pathogens. While a strong link has been documented between intestinal dysbiosis (i.e., disruption to the microbiota homeostasis) and diseases, the mechanisms by which commensal bacteria impact a wide spectrum of mucosal and extramucosal human disorders have only partially been deciphered. This is particularly puzzling for multiple myeloma (MM), a treatable but incurable neoplasia of plasma cells that accumulate in the bone marrow and lead to end-organ damage. Here we revise the most recent literature on data from both the bench and the bedside that show how the gut microbiota modulates cancer immunity, potentially impacting the progression of asymptomatic monoclonal gammopathy of undetermined significance (MGUS) and smoldering MM (SMM) to full blown MM. We also explore the effect of the gut microbiome on hematopoietic stem cell transplantation, chemotherapy, immunomodulating therapy and cancer immunotherapy in MM patients. Additionally, we identify the most cogent area of investigation that have the highest chance to delineate microbiota-related and pathobiology-based parameters for patient risk stratification. Lastly, we highlight microbiota-modulating strategies (i.e., diet, prebiotics, probiotics, fecal microbiota transplantation and postbiotics) that may reduce treatment-related toxicity in patients affected by MM as well as the rates of undertreatment of SMM patients.

## Introduction

The very moment we open our eyes to the world, our body has already been colonized by symbiotic microorganisms that will increase in number and species and become established through the first years of life into our own microbiota (1). This also is the time in which the immune system gets forged to recognize and eliminate pathogens (2) while acquiring tolerance to the self (3) and the host microbiota that becomes an extended self (4). Of note, children share a stereotypic immune system development that is microbiota-driven (5). A strong link between gut microbiota and immune dynamics is also found in adults undergoing immune reconstitution after hematopoietic stem cell transplantation (HSCT) (6). The mechanisms by which the microbiota interacts with the host immune system, and eventually modulates and/or gets modulated by the immune system is under investigation (7–30). The modulatory effects of the microbiota on the immune system are not only local, but also systemic and affect distant organs (31). Therefore, disruption to the microbiota homeostasis (i.e., dysbiosis) associates with diseases that span from allergy (32) and other immune-mediated diseases (33) to obesity (34), psychiatric disorders (35) and cancer (36). The microbiota also directly impacts human pathologies. As few examples, selected species of *Escherichia coli* alkylate DNA on adenosine residues and induce double strand breaks, eventually favoring mutations in colorectal cancer (37); intestinal commensal bacteria lead to androgen biosynthesis, thus promoting endocrine resistance in prostate cancer (38). Additionally, the gut microbiota influences susceptibility of cancer patients to surgery (39), chemotherapy (40), radiotherapy (41) and immunotherapy (42). Indeed, fecal microbiota transplant (FMT) from donors who achieved complete response to anti-PD-1 monotherapy into anti-PD-1-refractory melanoma patients resulted safe, feasible and associated with clinical responses and improved cancer control by the immune system (43). Modulation of the gut microbiome has also been attempted in patients affected by hematologic malignancies because the microbiota is highly susceptible to most of the treatments proposed to these patients (44), and microbiota translocation into the bloodstream of patients with therapy-induced immunosuppression contributes to morbidity and mortality (45). In turn, treatment-induced dysbiosis can be corrected by probiotics, prebiotics and FMT (46).

We focused here on multiple myeloma (MM), a treatable but incurable neoplasia of plasma cells that mainly accumulate in the bone marrow (BM) causing anemia, hypercalcemia, renal insufficiency, and bone lesions (47). Full blown MM is often preceded by two potentially curable asymptomatic diseases: monoclonal gammopathy of undetermined significance (MGUS) and smoldering MM (SMM) (48). Thus, identifying mechanisms by which MGUS and SMM patients progress to full-blown MM would represent a substantial clinical advancement. Microbiota-modulated immunity has been proposed as a mechanism of progression from SMM to MM (49). Additionally, several clinical and preclinical studies have highlighted the role of the gut microbiota in MM patients’ response to therapies (50, 51). Therefore, MM and its asymptomatic phases are examples of diseases in which alteration of the gut flora impacts disease progression, response to therapy and treatment-related toxicities. Information gathered in MM can be translated to other human diseases.

While we refer all interested readers to a more comprehensive review on this topic (52), in our short and more clinically-oriented paper we will start reporting data from both the bench and the bedside that show how the gut microbiota modulates MM. We will define the role of IL-17 in the crosstalk between MM and the intestinal microbiota. We will highlight how the gut microbiota is modified and can modify patients’ susceptibility to treatments. We will conclude with experimental and clinically strategies that can modulate the gut microbiota in patients affected by MGUS, SMM or MM.

## Gut Microbiota and Multiple Myeloma

Diet can profoundly affect the gut microbiota. While a link between diet an MM has been investigated for decades (53), only recently a correlation between microbiome and progression of MM has been searched for. As for other human diseases (4), the gut microbiota from MM patients has a reduced richness in bacterial species (54). *Bacteroides*, *Clostriudium leptum* and *Rothia* are enriched in MM patients when compared to family members, who usually share the microbiome (55). Interestingly, the level of *Clostridium leptum* positively correlates with ISS stage in MM patients. Because *Clostridium leptum* and *Rothia* are butyric acid-producing bacteria, the authors hypothesized dysregulation of the sugar metabolism in the intestine of MM patients (54). Whether short-chain fatty acids (SCFAs) have direct effects on neoplastic plasma cells or anti-MM immunity (56) needs to be investigated.

Opportunistic nitrogen-recycling bacteria such as *Klebsiella* and *Streptococcus* are enriched in the gut microbiota of MM patients (57). Accordingly, MM patients showed increased urease and glutamine synthetase activity in their feces and more urea and less ammonia in their blood than healthy subjects. Mice treated with FMT from MM patients and challenged with 5TGM1 MM cells experienced accelerated tumor burden that associated with elevated L-glutamine levels in their blood (57). Thus, gut colonization by nitrogen-recycling bacteria accelerates MM by making available L-glutamine. Building on this knowledge, fluoroglutamine might work as PET tracer in MM (58).

Altogether, these findings suggest that metabolites produced by a dysbiotic microbiota impact MM progression. Indeed, genetic diversity in the microbiome provides a wide variety of enzymes that convert polysaccharides and oligosaccharides into SCFAs like acetate, propionate, and butyrate (59). In mice, acetate, butyrate and pentanoate exert partially overlapping effects on T cells, B cells and dendritic cells (60, 61). By activating receptors expressed on intestinal epithelial cells and hematopoietic cells, SCFAs reduce inflammation (62, 63). In the context of autologous HSCT, the presence of the butyrate-producing bacteria *Eubacterium hallae* or *Faecalibacterium prausnitzii* in the gut microbiota of MM patients positively correlates with increased rates of minimal residual disease negativity (64). In rats, butyrate administration ameliorates colitis by increasing numbers of regulatory T cell (Treg) and suppressing levels of the pro-inflammatory cytokine IL-17A in both plasma and colonic mucosa (65). Conversely, commensals favoring the expansion of intestinal T helper-17 (Th17) cells may accelerate MM progression in mice (66). Thus, a strong link exists among commensal bacteria, Treg/Th17 cell balance and MM.

## IL-17 Swings the Balance Between Gut Microbiota and Multiple Myeloma

A different composition of the gut microbiota induces different immune responses locally and systemically (67). By producing IL-17, Th17 lymphocytes maintain a healthy intestinal mucosa and limit bacteria over-growth (68). Conversely, Treg cells allow tolerance to the extended self and limit excessive inflammation (69). Imbalance in the crosstalk between the host and the microbiota leads to excessive immune activation and expansion of Th17 cells.

Th17 cells are pathogenic in MM (70–72). Alexandrakis et al. (73) originally observed higher IL-17A levels in the peripheral blood of MM patients of stage II and III compared to stage I and a positive correlation with VEGF and microvessel density in the BM, thus hypothesizing a proangiogenic role for IL-17A in MM. Th17 cells are enriched in the BM of MM patients (70), where they support local inflammation and favor bone disease by promoting osteoclast differentiation (71). IL-17A, whose levels are increased in the BM of MM patients, contributes to neoplastic plasma cell survival and proliferation through the autocrine release of IL-6 (74). Also mouse neoplastic plasma cells express functional 17RA/RC (66), and *in vivo*, MM plasma cells upregulate cell proliferation and cell-cycle progression pathways if stimulated with IL-17A (75); while genes related to antigen processing and presentation and leukocyte trafficking and activation are downregulated in response to IL-17A (75). Neoplastic plasma cells can produce IL-17A, and treatment with antibodies specific for human IL-17A delayed growth of human MM in immunodeficient mice (76). These findings spurred a clinical trial with anti-IL-17A antibodies in MM patients (NCT03111992). IL-17A also resulted detrimental in the context of allogeneic-HSCT (77, 78). These works also identified IL-17A as the main path linking microbiota to Graft-Versus-Host Disease (GVHD). Indeed, in the absence of donor IL-17A, HSCT was more effective in controlling mouse MM (75).

While IL-17A is also elevated in some MGUS patients (79), a direct link has been reported between gut microbiota, IL-17A and progression of asymptomatic MM to full-blown MM (66). In Vk*MYC mice developing *de novo* MM that invariably evolves from asymptomatic to symptomatic MM (80), *Prevotella heparinolytica*, a human commensal (81), induces expansion of Th17 cells in the intestinal mucosa. Gut-born Th17 cells migrate to the BM, where they promote neoplastic plasma cell proliferation and progression from asymptomatic to symptomatic MM. At odds, *P. melaninogenica* restrain MM progression by limiting expansion of Th17 cells. Similarly, in SMM patients, high levels of BM IL-17 predicted faster progression to active MM (66). Lack of IL-17A in MM mice, or treatment with antibiotics or antibodies blocking IL-17/IL-17R interactions delayed disease progression (66). Thus, targeting the microbiota-IL17A axis in SMM patients might block disease progression.

## Role of the Gut Microbiota in Hematopoietic Stem Cell Transplantation

HSCT is a primary treatment for hematological malignancies and can be subdivided into autologous or allogeneic based on the use of self or donor-compatible HSCs, respectively (82). While the standard of care for MM patients is to receive high-dose chemotherapy followed by autologous-HSCT, allogeneic-HSCT can be proposed as part of a clinical trial and often associates with drawbacks like GVHD, a clinical condition in which the grafted immune system attacks tissues of the transplant recipient (83–85). The gut microbiota appears directly linked to allogeneic-HSCT success (86) and risk of GVHD (87). A large study including 111 MM patients across multiple clinical centers reported that lower diversity of intestinal microbiota associates with higher risk of transplant- and GHVD-related deaths (88).

Khan and colleagues highlighted interesting similarities in gut microbiota dysbiosis after both autologous- and allogeneic-HSCT in MM patients (89). Changes in the bacteriome and mycobiome also modulate early toxicity and the rate of neutrophil engraftment after autologous-HSCT (90). Generally, changes in bacterial abundances and species were linked to conditioning regimen or patient’s treatments (91, 92). Reduced bacterial diversity associates with increased immune activation, probability of relapse, GVHD and overall mortality (88, 91, 93–98). *Enterobacteria* and *Proteobacteria* abundance correlates with increasing probability of GVHD, pulmonary or gastro-intestinal complications and infection (92, 96, 98, 99). More in depth, enrichment in *Clostridium difficile* and *Rothia* associated with both autologous- and allogeneic-HSCT-related adverse events in MM patients (100), while colonization of species like *Akkermansia muciniphila* or *Enterococcus faecium* predispose to the dominance of other bacteria and further detrimental systemic consequences for patients (101). On the other hand, enrichment in *Ruminococcaceae*, *Lachnospiraceae* and *Clostridiales* correlates with higher transplantation efficiency and reduced GVHD (93, 102). Others identified *Blautia*, *Actinomyces*, *Prevotella* and *Eubacterium limosum* as commensals with protective effects (91, 98, 101).

Interestingly, a genus of bacteria may harbor species with opposing effects on the immune system (4). One example is *Clostridium difficile* that may increase risk of GVHD, disease relapse or mortality, contrary to other family members (6, 93, 99, 100). Different effects by different strains belonging to the same genus correlate to different metabolic activities. Butyrate and propionate can reduce GVHD, improve HSCT outcomes but also protect mice against radiation-induced injuries of the hematopoietic compartment (103). The microbiome also regulates energy uptake to improve allogeneic-HSCT outcomes in mice (97, 104), or lactose metabolism whose reduction, through diet or variation in microbial composition, limits pathological bacteria expansion and adverse effects in patients (105).

## Impact of the Gut Microbiota in Therapies for MM Patients

Because the gut microbiota can influence response to therapy and toxicity across different treatments (40), also in MM patients a link between gut microbiota and response to therapies has been investigated.

### Proteasome Inhibitors and Immunomodulating Drugs

MM patients benefit from combinations of proteasome inhibitors (PIs; bortezomib, carfilzomib, ixazomib) and immunomodulating drugs (IMiDs; thalidomide, lenalidomide, pomalidomide, dexamethasone) (106). PI treatment is burdened with gastrointestinal toxicity (107) that may also depend on gut microbiota (51). While the work by Pianko and colleagues did not investigate gastrointestinal toxicity in MM patients treated with PIs and/or IMiDs followed by autologous-HSCT, it showed an association between deep treatment response and enrichment in *Eubacterium hallii* and *Faecalibacterium prausnitzii* (64). *F. prausnitzii* usually associates with gut health, and both bacteria are SCFA producers (108), thus suggesting a potential link between SCFA producing bacteria a reduced gastrointestinal toxicity.

A higher prevalence of beneficial bacteria belonging to Bifidobacterium and Lactobacillus genus has been found in mice exposed to dexamethasone (109). Significantly decreased IL-17 levels in the intestinal mucosa and reduced colitis susceptibility were observed in mice receiving FMT from dexamethasone-conditioned donor mice (109). Thus, the immunosuppressive effect of dexamethasone may in part be supported by the induction of an anti-inflammatory microbiota (110).

### Chemotherapies

Cyclophosphamide (CTX) is an alkylating agent that stimulates type I interferon response and Th1/Th17 lymphocyte polarization (111). Because of the dual role of Th17 cells in MM pathogenesis and gut homeostasis, the effect of CTX on intestinal microbiota is relevant to understand the therapeutic outcomes in MM patients. *Bacteroidetes* and *Verrucomicrobia* are significantly reduced in mice treated with CTX (112). CTX treatment also increases intestinal barrier permeability and translocation of Gram-positive commensals, which favor Th1 and Th17 cell differentiation and anti-tumor immunity (113, 114).

Similarly, melphalan administration in rats causes severe injury to the small intestine, weight loss and infections (115). These effects recapitulate the toxicity observed in patients and limit melphalan administration to elderly subjects (116). Indeed, melphalan induces dysbiosis, reduction of SCFA production and bacterial translocation (115). Thus, replenishing SCFA or normalization of microbiota composition might re-establish intestinal homeostasis and improve drug tolerability.

### Cancer Immunotherapies

A strong correlation exists between gut microbiome and response to immune checkpoint inhibitors (ICIs) (43, 117–123). Interestingly, PD-L1 is expressed on malignant plasma cells, and PD-L1 predicts progression of SMM patients to MM (124). However, early-phase clinical trials with ICI as single agent showed modest activity in MM patients, whereas the combination with PIs ignited toxic events (125, 126). The latter might be linked to PI gastrointestinal toxicity (107). Andrews et al. associated defined microbiota signatures and abundance of *Bacteroides intestinalis* with upregulation of mucosal IL-1β and IL-17 in patients with ICI-related gastrointestinal adverse events (127). Administration of *Bifidobacterium* was sufficient to ameliorate ICI-related immunopathology in mice without dampening antitumor immunity (128). This strategy might also be adopted in MM patients.

The gut microbiota can promote the expansion and persistence of adoptively transferred cytotoxic T cells (CTLs) both in humans and mice (129). In mice, pentanoate and butyrate enhance anti-tumor activity of CTLs and chimeric antigen receptor (CAR)-T cells through metabolic and epigenetic reprogramming (130). A preliminary study on hematological patients receiving CAR-T cell therapy showed enrichment in *Ruminococcacaeae*, and *Lachnospiraceae*, which produce SCFA, in patients achieving complete remission (131). Because CAR-T cells are proposed to MM patients (132), it will be interesting to investigate how modulation of the gut microbiota can impact this treatment (133, 134).

## Conclusions

**Figure 1:**
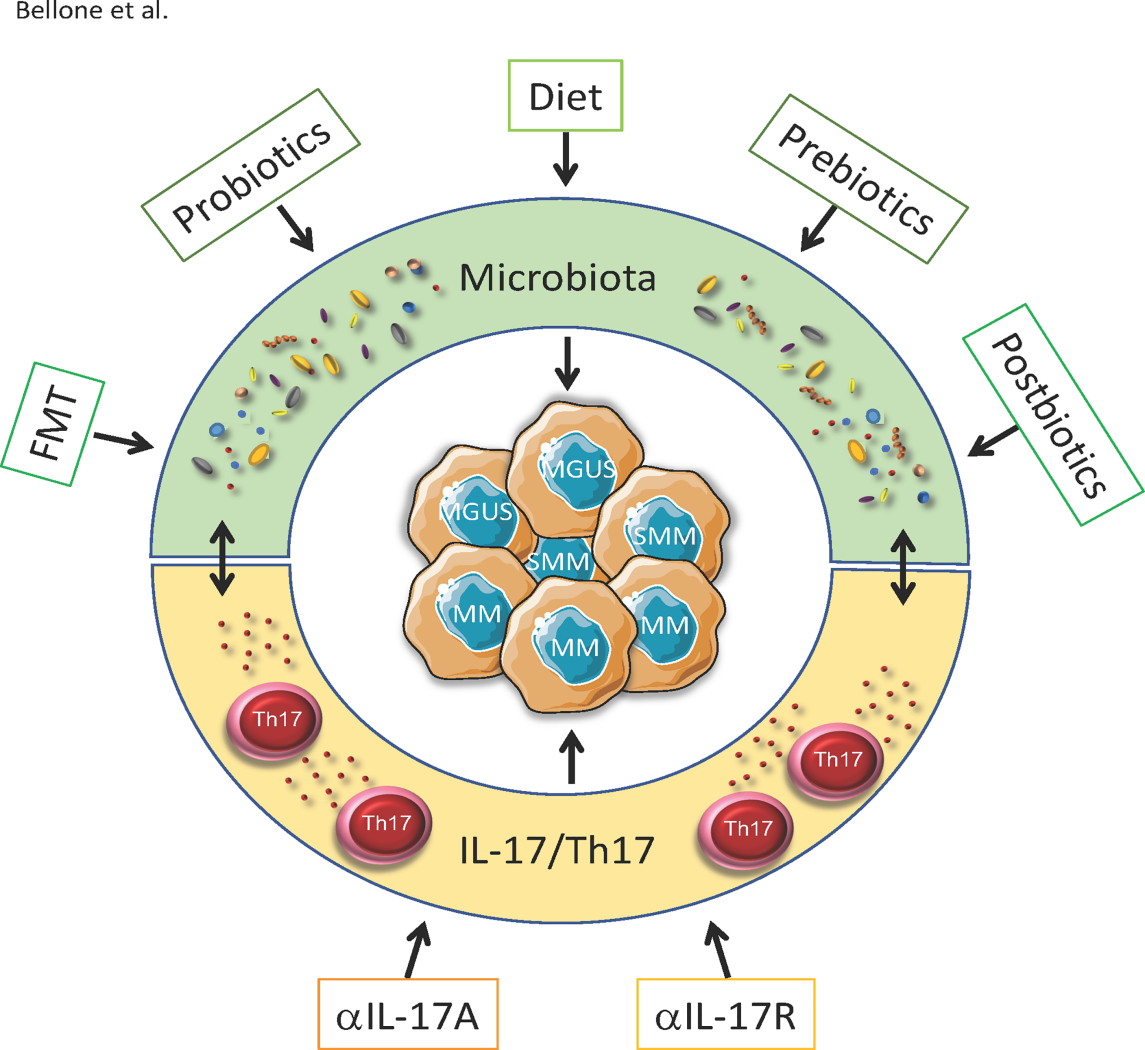
Strategies to impact MGUS, SMM and MM by targeting microbiota and IL-17/Th17. Progression from asymptomatic MGUS and SMM to symptomatic MM partially depends on the microbiota-Th17 axis. Peculiar composition of the gut microbiota locally induces the differentation of Th17 cells, which migrates in the BM and supports disease progression. Strategies to impact the disease by targeting the microbiota include diets enriched of specific nutrients(e.g.SCFA, vitamins, plant-specific foods etc.) autologous or heterologous FMT, prebiotics probiotic bacteria and postibiotics. All these strategies indirectly also reduce IL-17/Th17 accumulating in the gut and in the BM. Other than through the microbiota, monoclonal antibodies againstIL-17 and Il-17R interfere with its pathway and prevent the progression from SMM to MM in mice.

The information gathered so far strongly suggest that interfering with the insider (i.e., modifying the intestinal microbiota) may limit MM progression and increase susceptibility to therapies. These strategies include dietary intervention, administration of prebiotics, probiotics and postbiotics, but also FMT (**Figure 1**). While the concept of nutritional support is well established in the clinical practice (135), a more precise modulation of the gut microbiota has been attempted only recently, thanks to the acquisition of technologies for microbiome identification (36). Interestingly, unique microbial signatures can also be found in the peripheral blood within and between several cancer types (136).

Clinical trials aimed at modulating the gut microbiome to improve therapeutic response in hematopoietic malignancies are ongoing (**Table 1**) (137). Many of these trials propose administration of 1-6 different commensals with or without dietary intervention, and several of them focus on FMT. Major outcomes for patients undergoing HSCT are safety and GVHD control. Recently, a randomized trial has been launched to assess FMT efficacy in preventing allogeneic-HSCT complications in MM patients (NCT04935684). In other ongoing clinical trials, the microbiota is investigated in correlation to taste function (NCT03276481); to the supplementation of probiotic fermented milk product (NCT04530812); and to combined therapies (i.e., Selinixor, carfizomib and daratumumab or pomalidomide; NCT04661137). Many more studies deal with dietary intervention. As few examples, NCT04920084 will investigate whether a plant-rich diet is feasible and prevent MM in overweight individuals with MGUS or SMM. Another trial will determine if a specific mycobiome supporting diet can reduce gut inflammation in patients undergoing autologous-HSCT (NCT04685525). Resistant starch versus maltodextrin will be compared in a randomized trial involving candidates to autologous-HSCT (NCT05135351).

**Table 1:** Clinical trials in MM patients.

Trial ID	Patient population	Patients (n)	Intervention			Outcome	Result			Status (location)
Prebiotics										
NCT05135351	MM and Lymphoma patients undergoing autologous HSCT	30	Randomized, double-blind, placebo-controlled trial of resistant starch versus placebo			Primary: feasibility; secondary: hospital duration, rate of neutropenic fever, rate of broad-spectrum antibiotic exposure and rate of gastrointestinal symptoms	–			Not yet recruiting (University of Nebraska, USA)
NCT04629430	Patients affected by hematologic cancer and undergoing HSCT	29	Single group assignment, open label trial of prebiotics and HSCT			Primary: frequency of participants ingesting the required diet; secondary: incidence and severity of acute GVHD and acute GI GVHD, C. difficile infection, patient weight and days to neutrophil engraftment	–			Recruiting (University of Virginia, USA)
Probiotics										
NCT00946283	Patients undergoing donor allogenic HSCT for hematologic cancer or myelodysplastic syndrome	30	Single group assignment, open label trial of Lactobacillus rhamosus GG and HSCT			Primary: safety; secondary: none	Terminated due to slow accrual			Terminated (Rutgers Cancer Institute of New Jersey, USA)
NCT03057054	Patients undergoing alternative donor allogeneic HCT	500	Randomized, parallel assignment, placebo-controlled trial of Lactobacillus plantarum and HCT			Primary: Incidence of GI acute GVHD; secondary: none	–		Recruiting (Children’s Oncology Group, CA; National Cancer Institute, USA)	
NCT04530812	Asymptomatic MM patients	13	Randomized, parallel assignment, open label trial of Kefir and best practice		Primary: changes in biomarkers of metabolism, patient-reported pain, fatigue, gut health, and quality of life; Secondary: gut microbial community structure		–		Completed (Roswell Park Cancer Institute, USA)	
Diet										
NCT00003077	Advanced cancer patients who have significant weight loss and not amenable to curative therapy	63		Randomized, single group assignment, open label trial of high dose omega-3 fatty acids	Primary: survival; secondary: patient weight, maximum tolerated dose and antitumor response			Only 16% of patients has weight stabilization or weight gain	Completed (Holden Comprehensive Cancer Center, USA)	
NCT00469209	Primary refractory, relapsing after prior therapy MM patients	60		Randomized, parallel assignment, open label trial of vitamin c, arsenic trioxide, bortezomib and melphalan	Primary: toxicity and safety, efficacy and pharmacokinetics; secondary: time to toxicity			–	Completed (MD Anderson Cancer Center, USA)	
NCT00171925	MM and asymptomatic Stage I MM patients	143		Randomized, parallel assignment, open label trial of zolendronic acid, calcium and vitamin D	Primary: progression free survival; secondary: number of patients with skeletal-related events and complications			Reduced overall disease progression and skeletal events	Terminated (Novartis Investigative Site, DE)	
NCT00317811	MM and plasma cell neoplasm patients	35		Single arm, open label trial of ascorbic acid, bortezomib and melphalan	Primary: overall response, safety and tolerability, time to disease progression; secondary: time to response, PFS, OS			Disease controlled in 94% of patients	Completed (Oncotherapeutics, USA)	
NCT00661999	Anemic patients undergoing chemotherapy for nonmyeloid malignancies	502		Randomized, parallel assignment trial of ferrous sulfate, darbepoetin alfa and sodium ferric gluconate	Primary: hematopoietic response; secondary: hemoglobin levels, time to RBC transfusion, overall quality of life			No significant improvement	Completed (Mayo Clinic, USA)	
NCT00951626	Patients affected by hematologic cancer and undergoing allogenic HSCT	282		Randomized, parallel assignment trial of diet intervention	Primary: quality of life; secondary: time-to-complication, number of complications, mortality			–	Completed (City of Hope Comprehensive Cancer Center, USA)	
NCT04530812	MM patients	13		Randomized, parallel assignment, open labeled trial of kefir and best practice	Primary: biomarkers of metabolism, pain and fatigue, gut health, quality of life; secondary: gut microbial phylotype, bacterial metabolic function			–	Completed (Roswell Park Cancer Institute, USA)	
NCT04685525	MM patients undergoing autologous HSCT	40		Single group assignment, open labeled trial of mycobiome supporting diet	Primary: adherence; secondary: none			–	Not yet recruiting (University Hospitals Cleveland Medical Center, Case Comprehensive Cancer Center, USA)	
NCT04920084	MGUS, SMM patients	20		Single group assignment, open labeled trial of a plant-based diet	Primary: adherence, weight loss, BMI reduction; secondary: none			–	Recruiting (Memorial Sloan Kettering Cancer Center, USA)	
FMT										
NCT04935684	Patients affected by hematologic cancer and undergoing allogenic HSCT	150		Randomized, parallel assignment, open-label, multi-center trial of FMT	Primary: GVHD and RFS rate; secondary: OS, PFS, mortality			–	Not yet recruiting (University Hospital, Clermont-Ferrand, F)	

MM, multiple myeloma; SMM, smoldering multiple myeloma; HSCT, hematopoietic stem cell transplantation; GVHD, graft versus host disease; GI, gastrointestinal; C. difficile, Clostridium difficile; HCT, hematopoietic cell transplantation; PFS, progression free survival; OS, overall survival; RBC, red blood cells; BMI, body mass index; RFS, relapse free survival; FMT, fecal microbiota transplantation.

The natural tropism of bacteria for tumors have been exploited to design bacterial therapy for cancer (138). Commensals can be engineered to deliver drugs into tumors (36), and strategies have been devised to implement bacterial lysis with release of genetically encoded cargo within tumors only when a predefined population density of bacteria is reached (139–141).

Microbiota-derived metabolites (142) and microorganism-associated molecular patterns (143) can protect the BM from ionizing radiation toxicity. Additionally, recovery of lymphocytes and neutrophils after irradiation largely depends on gut microbiota, which also supports nutrients and caloric uptake (97). Because disruption of the intestinal microbiota occurs frequently in HSCT recipients as consequence of the conditioning regimens and wide-spectrum antibiotic use, peri-transplant treatment with simple nutrients, commensal-derived metabolites, selected bacterial species, or even FMT (144) should support optimal immune reconstitution and protection from GVHD and transplant-associated nutritional alterations (135). Of note, FMT in allogeneic-HCT recipients induced protection from intestinal GVHD that associated with increased abundance of butyrate-producing bacteria (144). Butyrate and propionate levels also associate with protection from chronic GVHD in patients affected by MM (145). Administration of resistant starch and prebiotics to allogeneic-HSCT recipients reduced the incidence of acute GVHD that associated with preservation of butyrate-producing commensals (146).

Modulation of the gut microbiota and its derivatives should be time and context dependent. While propionate can provide radioprotection (142), butyrate might limit the immunostimulatory activity of radiotherapy by decreasing dendritic cell antigen presentation (147). In the same vein, melanoma patients resistant to anti-CTLA-4 blockade showed high blood propionate and butyrate levels and higher proportion of Tregs (56). Thus, an excess of SCFAs, while protective in some contexts (60–63, 65, 93, 108), may be detrimental for the induction of efficient anti-cancer CTL responses (56, 147, 148). The situation is even more complex in MM patients, in which modulation of the gut microbiota and its metabolic derivatives should aim at restraining Th17 cell numbers in favor of optimal CTL responses (4). It will be interesting to investigate in animal models of MM if resistant fiber or SCFA supplementation in combination with immune checkpoint blockade limit the expansion of Th17 cells in favor of a potent anti-tumor immunity.

Manipulation of the gut microbiota is not without risks (149). Probiotic strains (e.g., *Lactobacilli*) can cause bacteriemia although the mechanism of transmission from probiotic to blood is unclear (150). Probiotics may also impair microbiota reconstitution after antibiotic-induced dysbiosis (151). Thus, further investigation is warranted to better understand the mechanistic links between prokaryotic and eukaryotic cells sharing our body space.

## Author Contributions

MB designed the review. MB, AB, LC, ML, and BM performed the literature review and wrote the manuscript. AB designed and created the table and the figure. All authors reviewed and edited the manuscript. All authors also approved the final version of the manuscript.

## Funding

The research leading to these results has received funding from Associazione Italiana per la Ricerca sul Cancro (AIRC) under IG2018- ID. 21808 to MB and was supported by a grant from the Leukemia & Lymphoma Society (# 6618-21) to MB. AB was supported by a fellowship from the Fondazione Italiana per la Ricerca sul Cancro/AIRC (Grants #22316 to AB).

## Conflict of Interest

Matteo Bellone and Arianna Brevi are co-owner of the following patent: WO2020/109620A2. bacterial strains for medical uses.

The remaining authors declare that the research was conducted in the absence of any commercial or financial relationships that could be construed as a potential conflict of interest.

## Publisher’s Note

All claims expressed in this article are solely those of the authors and do not necessarily represent those of their affiliated organizations, or those of the publisher, the editors and the reviewers. Any product that may be evaluated in this article, or claim that may be made by its manufacturer, is not guaranteed or endorsed by the publisher.

## References

[1] Gilbert JA Blaser MJ Caporaso JG Jansson JK Lynch SV Knight R. Current Understanding of the Human Microbiome. Nat Med (2018) 24:392–400. doi: 10.1038/nm.4517 29634682 PMC7043356

[2] Caballero S Pamer EG. Microbiota-Mediated Inflammation and Antimicrobial Defense in the Intestine. Annu Rev Immunol (2015) 33:227–56. doi: 10.1146/annurev-immunol-032713-120238 PMC4540477 25581310

[3] Bellone M. Autoimmune Disease: Pathogenesis. In: eLS Encyclopedia of Life Science. John Wiley & Sons, Ltd (Ed.). (2015). doi: 10.1002/9780470015902.a0001276.pub4

[4] Bellone M Brevi A Huber S. Microbiota-Propelled T Helper 17 Cells in Inflammatory Diseases and Cancer. Microbiol Mol Biol Rev (2020) 84(2):e00064–19. doi: 10.1128/MMBR.00064-19 PMC7062199 32132244

[5] Olin A Henckel E Chen Y Lakshmikanth T Pou C Mikes J. Stereotypic Immune System Development in Newborn Children. Cell (2018) 174:1277–92 e14. doi: 10.1016/j.cell.2018.06.045 30142345 PMC6108833

[6] Schluter J Peled JU Taylor BP Markey KA Smith M Taur Y. The Gut Microbiota is Associated With Immune Cell Dynamics in Humans. Nature (2020) 588:303–7. doi: 10.1038/s41586-020-2971-8 PMC7725892 33239790

[7] Cebula A Seweryn M Rempala GA Pabla SS McIndoe RA Denning TL. Thymus-Derived Regulatory T Cells Contribute to Tolerance to Commensal Microbiota. Nature (2013) 497:258–62. doi: 10.1038/nature12079 PMC3711137 23624374

[8] Esplugues E Huber S Gagliani N Hauser AE Town T Wan YY. Control of TH17 Cells Occurs in the Small Intestine. Nature (2011) 475:514–8. doi: 10.1038/nature10228 PMC3148838 21765430

[9] Lathrop SK Bloom SM Rao SM Nutsch K Lio CW Santacruz N. Peripheral Education of the Immune System by Colonic Commensal Microbiota. Nature (2011) 478:250–4. doi: 10.1038/nature10434 PMC3192908 21937990

[10] Nakajima A Negishi N Tsurui H Kadowaki-Ohtsuji N Maeda K Nanno M. Commensal Bacteria Regulate Thymic Aire Expression. PloS One (2014) 9:e105904. doi: 10.1371/journal.pone.0105904 25157574 PMC4144919

[11] Nutsch K Chai JN Ai TL Russler-Germain E Feehley T Nagler CR. Rapid and Efficient Generation of Regulatory T Cells to Commensal Antigens in the Periphery. Cell Rep (2016) 17:206–20. doi: 10.1016/j.celrep.2016.08.092 PMC5051580 27681432

[12] Sefik E Geva-Zatorsky N Oh S Konnikova L Zemmour D McGuire AM. Individual Intestinal Symbionts Induce a Distinct Population of RORgamma(+) Regulatory T Cells. Science (2015) 349:993–7. doi: 10.1126/science.aaa9420 PMC4700932 26272906

[13] Tanoue T Morita S Plichta DR Skelly AN Suda W Sugiura Y. A Defined Commensal Consortium Elicits CD8 T Cells and Anti-Cancer Immunity. Nature (2019) 565:600–5. doi: 10.1038/s41586-019-0878-z 30675064

[14] Geva-Zatorsky N Sefik E Kua L Pasman L Tan TG Ortiz-Lopez A. Mining the Human Gut Microbiota for Immunomodulatory Organisms. Cell (2017) 168:928–43 e11. doi: 10.1016/j.cell.2017.01.022 28215708 PMC7774263

[15] Depner M Taft DH Kirjavainen PV Kalanetra KM Karvonen AM Peschel S. Maturation of the Gut Microbiome During the First Year of Life Contributes to the Protective Farm Effect on Childhood Asthma. Nat Med (2020) 26:1766–75. doi: 10.1038/s41591-020-1095-x 33139948

[16] Nicholas DA Proctor EA Agrawal M Belkina AC Van Nostrand SC Panneerseelan-Bharath L. Fatty Acid Metabolites Combine With Reduced Beta Oxidation to Activate Th17 Inflammation in Human Type 2 Diabetes. Cell Metab (2019) 30:447–61.e5. doi: 10.1016/j.cmet.2019.07.004 31378464 PMC8506657

[17] Sullivan ZA Khoury-Hanold W Lim J Smillie C Biton M Reis BS. Gammadelta T Cells Regulate the Intestinal Response to Nutrient Sensing. Science (2021) 371(6535):eaba8310. doi: 10.1126/science.aba8310 33737460 PMC11617329

[18] Trompette A Gollwitzer ES Yadava K Sichelstiel AK Sprenger N Ngom-Bru C. Gut Microbiota Metabolism of Dietary Fiber Influences Allergic Airway Disease and Hematopoiesis. Nat Med (2014) 20:159–66. doi: 10.1038/nm.3444 24390308

[19] Yang W Yu T Huang X Bilotta AJ Xu L Lu Y. Intestinal Microbiota-Derived Short-Chain Fatty Acids Regulation of Immune Cell IL-22 Production and Gut Immunity. Nat Commun (2020) 11:4457. doi: 10.1038/s41467-020-18262-6 32901017 PMC7478978

[20] Sanchez HN Moroney JB Gan H Shen T Im JL Li T. B Cell-Intrinsic Epigenetic Modulation of Antibody Responses by Dietary Fiber-Derived Short-Chain Fatty Acids. Nat Commun (2020) 11:60. doi: 10.1038/s41467-019-13603-6 31896754 PMC6940392

[21] Garidou L Pomie C Klopp P Waget A Charpentier J Aloulou M. The Gut Microbiota Regulates Intestinal CD4 T Cells Expressing RORgammat and Controls Metabolic Disease. Cell Metab (2015) 22:100–12. doi: 10.1016/j.cmet.2015.06.001 26154056

[22] Marino E Richards JL McLeod KH Stanley D Yap YA Knight J. Gut Microbial Metabolites Limit the Frequency of Autoimmune T Cells and Protect Against Type 1 Diabetes. Nat Immunol (2017) 18:552–62. doi: 10.1038/ni.3713 28346408

[23] Schulthess J Pandey S Capitani M Rue-Albrecht KC Arnold I Franchini F. The Short Chain Fatty Acid Butyrate Imprints an Antimicrobial Program in Macrophages. Immunity (2019) 50:432–45.e7. doi: 10.1016/j.immuni.2018.12.018 30683619 PMC6382411

[24] Vogl T Klompus S Leviatan S Kalka IN Weinberger A Wijmenga C. Population-Wide Diversity and Stability of Serum Antibody Epitope Repertoires Against Human Microbiota. Nat Med (2021) 27:1442–50. doi: 10.1038/s41591-021-01409-3 34282338

[25] Berer K Mues M Koutrolos M Rasbi ZA Boziki M Johner C. Commensal Microbiota and Myelin Autoantigen Cooperate to Trigger Autoimmune Demyelination. Nature (2011) 479:538–41. doi: 10.1038/nature10554 22031325

[26] Wu HJ Ivanov II Darce J Hattori K Shima T Umesaki Y. Gut-Residing Segmented Filamentous Bacteria Drive Autoimmune Arthritis *via* T Helper 17 Cells. Immunity (2010) 32:815–27. doi: 10.1016/j.immuni.2010.06.001 PMC2904693 20620945

[27] Teng F Klinger CN Felix KM Bradley CP Wu E Tran NL. Gut Microbiota Drive Autoimmune Arthritis by Promoting Differentiation and Migration of Peyer’s Patch T Follicular Helper Cells. Immunity (2016) 44:875–88. doi: 10.1016/j.immuni.2016.03.013 PMC5296410 27096318

[28] Linehan JL Harrison OJ Han SJ Byrd AL Vujkovic-Cvijin I Villarino AV. Non-Classical Immunity Controls Microbiota Impact on Skin Immunity and Tissue Repair. Cell (2018) 172:784–96 e18. doi: 10.1016/j.cell.2017.12.033 29358051 PMC6034182

[29] Yang Y Torchinsky MB Gobert M Xiong H Xu M Linehan JL. Focused Specificity of Intestinal TH17 Cells Towards Commensal Bacterial Antigens. Nature (2014) 510:152–6. doi: 10.1038/nature13279 PMC4128479 24739972

[30] Zegarra-Ruiz DF Kim DV Norwood K Kim M Wu WH Saldana-Morales FB. Thymic Development of Gut-Microbiota-Specific T Cells. Nature (2021) 594:413–7. doi: 10.1038/s41586-021-03531-1 PMC8323488 33981034

[31] Brown EM Kenny DJ Xavier RJ. Gut Microbiota Regulation of T Cells During Inflammation and Autoimmunity. Annu Rev Immunol (2019) 37:599–624. doi: 10.1146/annurev-immunol-042718-041841 31026411

[32] Renz H Skevaki C. Early Life Microbial Exposures and Allergy Risks: Opportunities for Prevention. Nat Rev Immunol (2021) 21:177–91. doi: 10.1038/s41577-020-00420-y 32918062

[33] Ruff WE Greiling TM Kriegel MA. Host-Microbiota Interactions in Immune-Mediated Diseases. Nat Rev Microbiol (2020) 18:521–38. doi: 10.1038/s41579-020-0367-2 32457482

[34] Canfora EE Meex RCR Venema K Blaak EE. Gut Microbial Metabolites in Obesity, NAFLD and T2DM. Nat Rev Endocrinol (2019) 15:261–73. doi: 10.1038/s41574-019-0156-z 30670819

[35] Nikolova VL Smith MRB Hall LJ Cleare AJ Stone JM Young AH. Perturbations in Gut Microbiota Composition in Psychiatric Disorders: A Review and Meta-Analysis. JAMA Psychiatry (2021) 78(12):1343–54. doi: 10.1001/jamapsychiatry.2021.2573 PMC8444066 34524405

[36] Sepich-Poore GD Zitvogel L Straussman R Hasty J Wargo JA Knight R. The Microbiome and Human Cancer. Science (2021) 371. doi: 10.1126/science.abc4552 PMC8767999 33766858

[37] Pleguezuelos-Manzano C Puschhof J Rosendahl Huber A van Hoeck A Wood HM Nomburg J. Mutational Signature in Colorectal Cancer Caused by Genotoxic Pks(+) E. Coli Nature (2020) 580:269–73. doi: 10.1038/s41586-020-2080-8 PMC8142898 32106218

[38] Pernigoni N Zagato E Calcinotto A Troiani M Mestre RP Cali B. Commensal Bacteria Promote Endocrine Resistance in Prostate Cancer Through Androgen Biosynthesis. Science (2021) 374:216–24. doi: 10.1126/science.abf8403 34618582

[39] Guyton K Alverdy JC. The Gut Microbiota and Gastrointestinal Surgery. Nat Rev Gastroenterol Hepatol (2017) 14:43–54. doi: 10.1038/nrgastro.2016.139 27729657

[40] Roy S Trinchieri G. Microbiota: A Key Orchestrator of Cancer Therapy. Nat Rev Cancer (2017) 17:271–85. doi: 10.1038/nrc.2017.13 28303904

[41] Tonneau M Elkrief A Pasquier D Paz Del Socorro T Chamaillard M Bahig H. The Role of the Gut Microbiome on Radiation Therapy Efficacy and Gastrointestinal Complications: A Systematic Review. Radiother Oncol (2021) 156:1–9. doi: 10.1016/j.radonc.2020.10.033 33137398

[42] Derosa L Routy B Desilets A Daillere R Terrisse S Kroemer G. Microbiota-Centered Interventions: The Next Breakthrough in Immuno-Oncology? Cancer Discov (2021) 11:2396–412. doi: 10.1158/2159-8290.CD-21-0236 34400407

[43] Baruch EN Youngster I Ben-Betzalel G Ortenberg R Lahat A Katz L. Fecal Microbiota Transplant Promotes Response in Immunotherapy-Refractory Melanoma Patients. Science (2021) 371:602–9. doi: 10.1126/science.abb5920 33303685

[44] D’Angelo CR Sudakaran S Callander NS. Clinical Effects and Applications of the Gut Microbiome in Hematologic Malignancies. Cancer (2021) 127:679–87. doi: 10.1002/cncr.33400 PMC12805580 33369893

[45] Song Y Himmel B Ohrmalm L Gyarmati P. The Microbiota in Hematologic Malignancies. Curr Treat Options Oncol (2020) 21:2. doi: 10.1007/s11864-019-0693-7 31927673

[46] Severyn CJ Brewster R Andermann TM. Microbiota Modification in Hematology: Still at the Bench or Ready for the Bedside? Blood Advances (2019) 3:3461–72. doi: 10.1182/bloodadvances.2019000365 PMC6855120 31714965

[47] Palumbo A Anderson K. Multiple Myeloma. N Engl J Med (2011) 364:1046–60. doi: 10.1056/NEJMra1011442 21410373

[48] Kyle RA Larson DR Therneau TM Dispenzieri A Kumar S Cerhan JR. Long-Term Follow-Up of Monoclonal Gammopathy of Undetermined Significance. N Engl J Med (2018) 378:241–9. doi: 10.1056/NEJMoa1709974 PMC5852672 29342381

[49] Brevi A Cogrossi LL Grazia G Masciovecchio D Impellizzieri D Lacanfora L. Much More Than IL-17a: Cytokines of the IL-17 Family Between Microbiota and Cancer. Front Immunol (2020) 11:565470. doi: 10.3389/fimmu.2020.565470 33244315 PMC7683804

[50] Ahmed N Ghannoum M Gallogly M de Lima M Malek E. Influence of Gut Microbiome on Multiple Myeloma: Friend or Foe? J Immunother Cancer (2020) 8(1):e000576. doi: 10.1136/jitc-2020-000576 32581045 PMC7312329

[51] Alkharabsheh O Sidiqi MH Aljama MA Gertz MA Frankel AE. The Human Microbiota in Multiple Myeloma and Proteasome Inhibitors. Acta Haematol (2020) 143:118–23. doi: 10.1159/000500976 31311009

[52] Jasinski M Bilinski J Basak GW. The Role of the Gut Microbiome in Pathogenesis, Biology, and Treatment of Plasma Cell Dyscrasias. Front Oncol (2021) 11:741376. doi: 10.3389/fonc.2021.741376 34660303 PMC8517391

[53] Shapiro YN Peppercorn JM Yee AJ Branagan AR Raje NS Donnell EKO. Lifestyle Considerations in Multiple Myeloma. Blood Cancer J (2021) 11:172. doi: 10.1038/s41408-021-00560-x 34702799 PMC8548591

[54] Zhang B Gu J Liu J Huang B Li J. Fecal Microbiota Taxonomic Shifts in Chinese Multiple Myeloma Patients Analyzed by Quantitative Polimerase Chain Reaction (QPCR) and 16S rRNA High-Throughput Sequencing. Med Sci Monit (2019) 25:8269–80. doi: 10.12659/MSM.919988 PMC6855177 31678982

[55] Lax S Smith DP Hampton-Marcell J Owens SM Handley KM Scott NM. Longitudinal Analysis of Microbial Interaction Between Humans and the Indoor Environment. Science (2014) 345:1048–52. doi: 10.1126/science.1254529 PMC4337996 25170151

[56] Coutzac C Jouniaux JM Paci A Schmidt J Mallardo D Seck A. Systemic Short Chain Fatty Acids Limit Antitumor Effect of CTLA-4 Blockade in Hosts With Cancer. Nat Commun (2020) 11:2168. doi: 10.1038/s41467-020-16079-x 32358520 PMC7195489

[57] Jian X Zhu Y Ouyang J Wang Y Lei Q Xia J. Alterations of Gut Microbiome Accelerate Multiple Myeloma Progression by Increasing the Relative Abundances of Nitrogen-Recycling Bacteria. Microbiome (2020) 8:74. doi: 10.1186/s40168-020-00854-5 32466801 PMC7257554

[58] Valtorta S Toscani D Chiu M Sartori A Coliva A Brevi A. [(18)F](2S,4R)-4-Fluoroglutamine as a New Positron Emission Tomography Tracer in Myeloma. Front Oncol (2021) 11:760732. doi: 10.3389/fonc.2021.760732 34712616 PMC8546185

[59] Honda K Littman DR. The Microbiome in Infectious Disease and Inflammation. Annu Rev Immunol (2012) 30:759–95. doi: 10.1146/annurev-immunol-020711-074937 PMC4426968 22224764

[60] Brevi A Bellone M. Fatty is Not That Bad: Feeding Short-Chain Fatty Acids to Restrain Autoimmunity. Cell Mol Immunol (2017) 14(11):878–80. doi: 10.1038/cmi.2017.52 PMC5675955 28713165

[61] Luu M Pautz S Kohl V Singh R Romero R Lucas S. The Short-Chain Fatty Acid Pentanoate Suppresses Autoimmunity by Modulating the Metabolic-Epigenetic Crosstalk in Lymphocytes. Nat Commun (2019) 10:760. doi: 10.1038/s41467-019-08711-2 30770822 PMC6377655

[62] Maslowski KM Mackay CR. Diet, Gut Microbiota and Immune Responses. Nat Immunol (2011) 12:5–9. doi: 10.1038/ni0111-5 21169997

[63] Macia L Tan J Vieira AT Leach K Stanley D Luong S. Metabolite-Sensing Receptors GPR43 and GPR109A Facilitate Dietary Fibre-Induced Gut Homeostasis Through Regulation of the Inflammasome. Nat Commun (2015) 6:6734. doi: 10.1038/ncomms7734 25828455

[64] Pianko MJ Devlin SM Littmann ER Chansakul A Mastey D Salcedo M. Minimal Residual Disease Negativity in Multiple Myeloma Is Associated With Intestinal Microbiota Composition. Blood Advances (2019) 3:2040–4. doi: 10.1182/bloodadvances.2019032276 PMC6616258 31289031

[65] Zhang M Zhou Q Dorfman RG Huang X Fan T Zhang H. Butyrate Inhibits Interleukin-17 and Generates Tregs to Ameliorate Colorectal Colitis in Rats. BMC Gastroenterol (2016) 16:84. doi: 10.1186/s12876-016-0500-x 27473867 PMC4967301

[66] Calcinotto A Brevi A Chesi M Ferrarese R Garcia Perez L Grioni M. Microbiota-Driven Interleukin-17-Producing Cells and Eosinophils Synergize to Accelerate Multiple Myeloma Progression. Nat Commun (2018) 9:4832. doi: 10.1038/s41467-018-07305-8 30510245 PMC6277390

[67] Kundu P Blacher E Elinav E Pettersson S. Our Gut Microbiome: The Evolving Inner Self. Cell (2017) 171:1481–93. doi: 10.1016/j.cell.2017.11.024 29245010

[68] Macpherson AJ Uhr T. Induction of Protective IgA by Intestinal Dendritic Cells Carrying Commensal Bacteria. Science (2004) 303:1662–5. doi: 10.1126/science.1091334 15016999

[69] Tanoue T Atarashi K Honda K. Development and Maintenance of Intestinal Regulatory T Cells. Nat Rev Immunol (2016) 16:295–309. doi: 10.1038/nri.2016.36 27087661

[70] Dhodapkar KM Barbuto S Matthews P Kukreja A Mazumder A Vesole D. Dendritic Cells Mediate the Induction of Polyfunctional Human IL17-Producing Cells (Th17-1 Cells) Enriched in the Bone Marrow of Patients With Myeloma. Blood (2008) 112:2878–85. doi: 10.1182/blood-2008-03-143222 PMC2556623 18669891

[71] Noonan K Marchionni L Anderson J Pardoll D Roodman GD Borrello I. A Novel Role of IL-17-Producing Lymphocytes in Mediating Lytic Bone Disease in Multiple Myeloma. Blood (2010) 116:3554–63. doi: 10.1182/blood-2010-05-283895 PMC4017298 20664052

[72] Bryant C Suen H Brown R Yang S Favaloro J Aklilu E. Long-Term Survival in Multiple Myeloma is Associated With a Distinct Immunological Profile, Which Includes Proliferative Cytotoxic T-Cell Clones and a Favourable Treg/Th17 Balance. Blood Cancer J (2013) 3:e148. doi: 10.1038/bcj.2013.34 24036947 PMC3789202

[73] Alexandrakis MG Pappa CA Miyakis S Sfiridaki A Kafousi M Alegakis A. Serum Interleukin-17 and its Relationship to Angiogenic Factors in Multiple Myeloma. Eur J Intern Med (2006) 17:412–6. doi: 10.1016/j.ejim.2006.02.012 16962948

[74] Prabhala RH Pelluru D Fulciniti M Prabhala HK Nanjappa P Song W. Elevated IL-17 Produced by TH17 Cells Promotes Myeloma Cell Growth and Inhibits Immune Function in Multiple Myeloma. Blood (2010) 115:5385–92. doi: 10.1182/blood-2009-10-246660 PMC2902136 20395418

[75] Vuckovic S Minnie SA Smith D Gartlan KH Watkins TS Markey KA. Bone Marrow Transplantation Generates T Cell-Dependent Control of Myeloma in Mice. J Clin Invest (2019) 129:106–21. doi: 10.1172/JCI98888 PMC6307976 30300141

[76] Prabhala RH Fulciniti M Pelluru D Rashid N Nigroiu A Nanjappa P. Targeting IL-17A in Multiple Myeloma: A Potential Novel Therapeutic Approach in Myeloma. Leukemia (2016) 30:379–89. doi: 10.1038/leu.2015.228 PMC4740263 26293646

[77] Kappel LW Goldberg GL King CG Suh DY Smith OM Ligh C. IL-17 Contributes to CD4-Mediated Graft-Versus-Host Disease. Blood (2009) 113:945–52. doi: 10.1182/blood-2008-08-172155 PMC2630280 18931341

[78] Varelias A Ormerod KL Bunting MD Koyama M Gartlan KH Kuns RD. Acute Graft-Versus-Host Disease is Regulated by an IL-17-Sensitive Microbiome. Blood (2017) 129:2172–85. doi: 10.1182/blood-2016-08-732628 PMC5391622 28137828

[79] Bosseboeuf A Allain-Maillet S Mennesson N Tallet A Rossi C Garderet L. Pro-Inflammatory State in Monoclonal Gammopathy of Undetermined Significance and in Multiple Myeloma Is Characterized by Low Sialylation of Pathogen-Specific and Other Monoclonal Immunoglobulins. Front Immunol (2017) 8:1347. doi: 10.3389/fimmu.2017.01347 29098000 PMC5653692

[80] Calcinotto A Grioni M Jachetti E Curnis F Mondino A Parmiani G. Targeting TNF-Alpha to Neoangiogenic Vessels Enhances Lymphocyte Infiltration in Tumors and Increases the Therapeutic Potential of Immunotherapy. J Immunol (2012) 188:2687–94. doi: 10.4049/jimmunol.1101877 22323546

[81] Arumugam M Raes J Pelletier E Le Paslier D Yamada T Mende DR. Enterotypes of the Human Gut Microbiome. Nature (2011) 473:174–80. doi: 10.1038/nature09944 PMC3728647 21508958

[82] Blazar BR Hill GR Murphy WJ. Dissecting the Biology of Allogeneic HSCT to Enhance the GvT Effect Whilst Minimizing GvHD. Nat Rev Clin Oncol (2020) 17:475–92. doi: 10.1038/s41571-020-0356-4 PMC7901860 32313224

[83] Bjorkstrand BB Ljungman P Svensson H Hermans J Alegre A Apperley J. Allogeneic Bone Marrow Transplantation Versus Autologous Stem Cell Transplantation in Multiple Myeloma: A Retrospective Case-Matched Study From the European Group for Blood and Marrow Transplantation. Blood (1996) 88:4711–8. doi: 10.1182/blood.V88.12.4711.bloodjournal88124711 8977265

[84] Bruno B Rotta M Patriarca F Mordini N Allione B Carnevale-Schianca F. A Comparison of Allografting With Autografting for Newly Diagnosed Myeloma. N Engl J Med (2007) 356:1110–20. doi: 10.1056/NEJMoa065464 17360989

[85] Giralt S Costa LJ Maloney D Krishnan A Fei M Antin JH. Tandem Autologous-Autologous Versus Autologous-Allogeneic Hematopoietic Stem Cell Transplant for Patients With Multiple Myeloma: Long-Term Follow-Up Results From the Blood and Marrow Transplant Clinical Trials Network 0102 Trial. Biol Blood Marrow Transplant (2020) 26:798–804. doi: 10.1016/j.bbmt.2019.11.018 31756536 PMC7198329

[86] Shono Y van den Brink MRM. Gut Microbiota Injury in Allogeneic Haematopoietic Stem Cell Transplantation. Nat Rev Cancer (2018) 18:283–95. doi: 10.1038/nrc.2018.10 PMC7485905 29449660

[87] Fredricks DN. The Gut Microbiota and Graft-Versus-Host Disease. J Clin Invest (2019) 129:1808–17. doi: 10.1172/JCI125797 PMC6486325 31042160

[88] Peled JU Gomes ALC Devlin SM Littmann ER Taur Y Sung AD. Microbiota as Predictor of Mortality in Allogeneic Hematopoietic-Cell Transplantation. N Engl J Med (2020) 382:822–34. doi: 10.1056/NEJMoa1900623 PMC7534690 32101664

[89] Khan N Lindner S Gomes ALC Devlin SM Shah GL Sung AD. Fecal Microbiota Diversity Disruption and Clinical Outcomes After Auto-HCT: A Multicenter Observational Study. Blood (2021) 137:1527–37. doi: 10.1182/blood.2020006923 PMC7976512 33512409

[90] El Jurdi N Filali-Mouhim A Salem I Retuerto M Dambrosio NM Baer L. Gastrointestinal Microbiome and Mycobiome Changes During Autologous Transplantation for Multiple Myeloma: Results of a Prospective Pilot Study. Biol Blood Marrow Transplant (2019) 25:1511–9. doi: 10.1016/j.bbmt.2019.04.007 30959164

[91] Peled JU Devlin SM Staffas A Lumish M Khanin R Littmann ER. Intestinal Microbiota and Relapse After Hematopoietic-Cell Transplantation. J Clin Oncol (2017) 35:1650–9. doi: 10.1200/JCO.2016.70.3348 PMC5455763 28296584

[92] Rafei H Jenq RR. Microbiome-Intestine Cross Talk During Acute Graft-Versus-Host Disease. Blood (2020) 136:401–9. doi: 10.1182/blood.2019000950 PMC7378453 32526029

[93] Jenq RR Ubeda C Taur Y Menezes CC Khanin R Dudakov JA. Regulation of Intestinal Inflammation by Microbiota Following Allogeneic Bone Marrow Transplantation. J Exp Med (2012) 209:903–11. doi: 10.1084/jem.20112408 PMC3348096 22547653

[94] Taur Y Jenq RR Perales MA Littmann ER Morjaria S Ling L. The Effects of Intestinal Tract Bacterial Diversity on Mortality Following Allogeneic Hematopoietic Stem Cell Transplantation. Blood (2014) 124:1174–82. doi: 10.1182/blood-2014-02-554725 PMC4133489 24939656

[95] Harris B Morjaria SM Littmann ER Geyer AI Stover DE Barker JN. Gut Microbiota Predict Pulmonary Infiltrates After Allogeneic Hematopoietic Cell Transplantation. Am J Respir Crit Care Med (2016) 194:450–63. doi: 10.1164/rccm.201507-1491OC PMC5003327 26886180

[96] Mancini N Greco R Pasciuta R Barbanti MC Pini G Morrow OB. Enteric Microbiome Markers as Early Predictors of Clinical Outcome in Allogeneic Hematopoietic Stem Cell Transplant: Results of a Prospective Study in Adult Patients. Open Forum Infect Dis (2017) 4:ofx215. doi: 10.1093/ofid/ofx215 29226172 PMC5714175

[97] Staffas A Burgos da Silva M Slingerland AE Lazrak A Bare CJ Holman CD. Nutritional Support From the Intestinal Microbiota Improves Hematopoietic Reconstitution After Bone Marrow Transplantation in Mice. Cell Host Microbe (2018) 23:447–57 e4. doi: 10.1016/j.chom.2018.03.002 29576480 PMC5897172

[98] Greco R Nitti R Mancini N Pasciuta R Lorentino F Lupo-Stanghellini MT. Microbiome Markers are Early Predictors of Acute GVHD in Allogeneic Hematopoietic Stem Cell Transplant Recipients. Blood (2021) 137:1556–9. doi: 10.1182/blood.2020007158 33171492

[99] Holler E Butzhammer P Schmid K Hundsrucker C Koestler J Peter K. Metagenomic Analysis of the Stool Microbiome in Patients Receiving Allogeneic Stem Cell Transplantation: Loss of Diversity is Associated With Use of Systemic Antibiotics and More Pronounced in Gastrointestinal Graft-Versus-Host Disease. Biol Blood Marrow Transplant (2014) 20:640–5. doi: 10.1016/j.bbmt.2014.01.030 PMC4973578 24492144

[100] Alonso CD Treadway SB Hanna DB Huff CA Neofytos D Carroll KC. Epidemiology and Outcomes of Clostridium Difficile Infections in Hematopoietic Stem Cell Transplant Recipients. Clin Infect Dis (2012) 54:1053–63. doi: 10.1093/cid/cir1035 PMC3309884 22412059

[101] Taur Y Xavier JB Lipuma L Ubeda C Goldberg J Gobourne A. Intestinal Domination and the Risk of Bacteremia in Patients Undergoing Allogeneic Hematopoietic Stem Cell Transplantation. Clin Infect Dis (2012) 55:905–14. doi: 10.1093/cid/cis580 PMC3657523 22718773

[102] Weber D Oefner PJ Hiergeist A Koestler J Gessner A Weber M. Low Urinary Indoxyl Sulfate Levels Early After Transplantation Reflect a Disrupted Microbiome and are Associated With Poor Outcome. Blood (2015) 126:1723–8. doi: 10.1182/blood-2015-04-638858 26209659

[103] Mathewson ND Jenq R Mathew AV Koenigsknecht M Hanash A Toubai T. Gut Microbiome-Derived Metabolites Modulate Intestinal Epithelial Cell Damage and Mitigate Graft-Versus-Host Disease. Nat Immunol (2016) 17:505–13. doi: 10.1038/ni.3400 PMC4836986 26998764

[104] Backhed F Ding H Wang T Hooper LV Koh GY Nagy A. The Gut Microbiota as an Environmental Factor That Regulates Fat Storage. Proc Natl Acad Sci USA (2004) 101:15718–23. doi: 10.1073/pnas.0407076101 PMC524219 15505215

[105] Stein-Thoeringer CK Nichols KB Lazrak A Slingerland AE Slingerland JB. Lactose Drives Enterococcus Expansion to Promote Graft-Versus-Host Disease. Science (2019) 366:1143–9. doi: 10.1126/science.aax3760 PMC7003985 31780560

[106] Rajkumar SV Kumar S. Multiple Myeloma Current Treatment Algorithms. Blood Cancer J (2020) 10:94. doi: 10.1038/s41408-020-00359-2 32989217 PMC7523011

[107] Stansborough RL Gibson RJ. Proteasome Inhibitor-Induced Gastrointestinal Toxicity. Curr Opin Support Palliat Care (2017) 11:133–7. doi: 10.1097/SPC.0000000000000266 28333868

[108] Ferreira-Halder CV Faria AVS Andrade SS. Action and Function of Faecalibacterium Prausnitzii in Health and Disease. Best Pract Res Clin Gastroenterol (2017) 31:643–8. doi: 10.1016/j.bpg.2017.09.011 29566907

[109] Huang EY Inoue T Leone VA Dalal S Touw K Wang Y. Using Corticosteroids to Reshape the Gut Microbiome: Implications for Inflammatory Bowel Diseases. Inflammation Bowel Dis (2015) 21:963–72. doi: 10.1097/MIB.0000000000000332 PMC4402247 25738379

[110] Luo XM Edwards MR Mu Q Yu Y Vieson MD Reilly CM. Gut Microbiota in Human Systemic Lupus Erythematosus and a Mouse Model of Lupus. Appl Environ Microbiol (2018) 84(4):e02288–17. doi: 10.1128/AEM.02288-17 PMC5795066 29196292

[111] Sistigu A Viaud S Chaput N Bracci L Proietti E Zitvogel L. Immunomodulatory Effects of Cyclophosphamide and Implementations for Vaccine Design. Semin Immunopathol (2011) 33:369–83. doi: 10.1007/s00281-011-0245-0 21611872

[112] Xu X Zhang X. Effects of Cyclophosphamide on Immune System and Gut Microbiota in Mice. Microbiol Res (2015) 171:97–106. doi: 10.1016/j.micres.2014.11.002 25553830

[113] Viaud S Saccheri F Mignot G Yamazaki T Daillere R Hannani D. The Intestinal Microbiota Modulates the Anticancer Immune Effects of Cyclophosphamide. Science (2013) 342:971–6. doi: 10.1126/science.1240537 PMC4048947 24264990

[114] Yang J Liu KX Qu JM Wang XD. The Changes Induced by Cyclophosphamide in Intestinal Barrier and Microflora in Mice. Eur J Pharmacol (2013) 714:120–4. doi: 10.1016/j.ejphar.2013.06.006 23791611

[115] Wardill HR de Mooij CEM da Silva Ferreira AR van de Peppel IP Havinga R Harmsen HJM. Translational Model of Melphalan-Induced Gut Toxicity Reveals Drug-Host-Microbe Interactions That Drive Tissue Injury and Fever. Cancer Chemother Pharmacol (2021) 88:173–88. doi: 10.1007/s00280-021-04273-7 PMC8236460 33877390

[116] van der Velden WJ Herbers AH Feuth T Schaap NP Donnelly JP Blijlevens NM. Intestinal Damage Determines the Inflammatory Response and Early Complications in Patients Receiving Conditioning for a Stem Cell Transplantation. PloS One (2010) 5:e15156. doi: 10.1371/journal.pone.0015156 21188146 PMC3004799

[117] Vetizou M Pitt JM Daillere R Lepage P Waldschmitt N Flament C. Anticancer Immunotherapy by CTLA-4 Blockade Relies on the Gut Microbiota. Science (2015) 350:1079–84. doi: 10.1126/science.aad1329 PMC4721659 26541610

[118] Sivan A Corrales L Hubert N Williams JB Aquino-Michaels K Earley ZM. Commensal Bifidobacterium Promotes Antitumor Immunity and Facilitates Anti-PD-L1 Efficacy. Science (2015) 350:1084–9. doi: 10.1126/science.aac4255 PMC4873287 26541606

[119] Gopalakrishnan V Spencer CN Nezi L Reuben A Andrews MC Karpinets TV. Gut Microbiome Modulates Response to Anti-PD-1 Immunotherapy in Melanoma Patients. Science (2018) 359:97–103. doi: 10.1126/science.aan4236 29097493 PMC5827966

[120] Matson V Fessler J Bao R Chongsuwat T Zha Y Alegre ML. The Commensal Microbiome is Associated With Anti-PD-1 Efficacy in Metastatic Melanoma Patients. Science (2018) 359:104–8. doi: 10.1126/science.aao3290 PMC6707353 29302014

[121] Routy B Le Chatelier E Derosa L Duong CPM Alou MT Daillere R. Gut Microbiome Influences Efficacy of PD-1-Based Immunotherapy Against Epithelial Tumors. Science (2018) 359:91–7. doi: 10.1126/science.aan3706 29097494

[122] Mager LF Burkhard R Pett N Cooke NCA Brown K Ramay H. Microbiome-Derived Inosine Modulates Response to Checkpoint Inhibitor Immunotherapy. Science (2020) 369:1481–9. doi: 10.1126/science.abc3421 32792462

[123] Davar D Dzutsev AK McCulloch JA Rodrigues RR Chauvin JM Morrison RM. Fecal Microbiota Transplant Overcomes Resistance to Anti-PD-1 Therapy in Melanoma Patients. Science (2021) 371:595–602. doi: 10.1126/science.abf3363 33542131 PMC8097968

[124] Gorgun G Samur MK Cowens KB Paula S Bianchi G Anderson JE. Lenalidomide Enhances Immune Checkpoint Blockade-Induced Immune Response in Multiple Myeloma. Clin Cancer Res (2015) 21:4607–18. doi: 10.1158/1078-0432.CCR-15-0200 PMC4609232 25979485

[125] Jelinek T Paiva B Hajek R. Update on PD-1/PD-L1 Inhibitors in Multiple Myeloma. Front Immunol (2018) 9:2431. doi: 10.3389/fimmu.2018.02431 30505301 PMC6250817

[126] Lesokhin AM Bal S Badros AZ. Lessons Learned From Checkpoint Blockade Targeting PD-1 in Multiple Myeloma. Cancer Immunol Res (2019) 7:1224–9. doi: 10.1158/2326-6066.CIR-19-0148 PMC6891823 31371317

[127] Andrews MC Duong CPM Gopalakrishnan V Iebba V Chen WS Derosa L. Gut Microbiota Signatures are Associated With Toxicity to Combined CTLA-4 and PD-1 Blockade. Nat Med (2021) 27:1432–41. doi: 10.1038/s41591-021-01406-6 PMC11107795 34239137

[128] Wang F Yin Q Chen L Davis MM. Bifidobacterium can Mitigate Intestinal Immunopathology in the Context of CTLA-4 Blockade. Proc Natl Acad Sci USA (2018) 115:157–61. doi: 10.1073/pnas.1712901115 PMC5776803 29255057

[129] He Y Fu L Li Y Wang W Gong M Zhang J. Gut Microbial Metabolites Facilitate Anticancer Therapy Efficacy by Modulating Cytotoxic CD8(+) T Cell Immunity. Cell Metab (2021) 33:988–1000 e7. doi: 10.1016/j.cmet.2021.03.002 33761313

[130] Luu M Riester Z Baldrich A Reichardt N Yuille S Busetti A. Microbial Short-Chain Fatty Acids Modulate CD8(+) T Cell Responses and Improve Adoptive Immunotherapy for Cancer. Nat Commun (2021) 12:4077. doi: 10.1038/s41467-021-24331-1 34210970 PMC8249424

[131] Smith M Zakrzewski J James S Sadelain M. Posttransplant Chimeric Antigen Receptor Therapy. Blood (2018) 131:1045–52. doi: 10.1182/blood-2017-08-752121 PMC5865610 29358181

[132] van de Donk N Usmani SZ Yong K. CAR T-Cell Therapy for Multiple Myeloma: State of the Art and Prospects. Lancet Haematol (2021) 8:e446–e61. doi: 10.1016/S2352-3026(21)00057-0 34048683

[133] Schubert ML Rohrbach R Schmitt M Stein-Thoeringer CK. The Potential Role of the Intestinal Micromilieu and Individual Microbes in the Immunobiology of Chimeric Antigen Receptor T-Cell Therapy. Front Immunol (2021) 12:670286. doi: 10.3389/fimmu.2021.670286 34135898 PMC8200823

[134] Di Nicola M Apetoh L Bellone M Colombo MP Dotti G Ferrone S. Innovative Therapy, Monoclonal Antibodies and Beyond. Cytokine Growth Factor Rev (2017) 38:1–9. doi: 10.1016/j.cytogfr.2017.10.002 29029813

[135] Baumgartner A Bargetzi A Zueger N Bargetzi M Medinger M Bounoure L. Revisiting Nutritional Support for Allogeneic Hematologic Stem Cell Transplantation-a Systematic Review. Bone Marrow Transplant (2017) 52:506–13. doi: 10.1038/bmt.2016.310 28067888

[136] Poore GD Kopylova E Zhu Q Carpenter C Fraraccio S Wandro S. Microbiome Analyses of Blood and Tissues Suggest Cancer Diagnostic Approach. Nature (2020) 579:567–74. doi: 10.1038/s41586-020-2095-1 PMC7500457 32214244

[137] McQuade JL Daniel CR Helmink BA Wargo JA. Modulating the Microbiome to Improve Therapeutic Response in Cancer. Lancet Oncol (2019) 20:e77–91. doi: 10.1016/S1470-2045(18)30952-5 PMC12908161 30712808

[138] Zheng JH Nguyen VH Jiang SN Park SH Tan W Hong SH. Two-Step Enhanced Cancer Immunotherapy With Engineered Salmonella Typhimurium Secreting Heterologous Flagellin. Sci Transl Med (2017) 9(376):eaak9537. doi: 10.1126/scitranslmed.aak9537 28179508

[139] Swofford CA Van Dessel N Forbes NS. Quorum-Sensing Salmonella Selectively Trigger Protein Expression Within Tumors. Proc Natl Acad Sci USA (2015) 112:3457–62. doi: 10.1073/pnas.1414558112 PMC4371937 25737556

[140] Din MO Danino T Prindle A Skalak M Selimkhanov J Allen K. Synchronized Cycles of Bacterial Lysis for *In Vivo* Delivery. Nature (2016) 536:81–5. doi: 10.1038/nature18930 PMC5048415 27437587

[141] Chowdhury S Castro S Coker C Hinchliffe TE Arpaia N Danino T. Programmable Bacteria Induce Durable Tumor Regression and Systemic Antitumor Immunity. Nat Med (2019) 25:1057–63. doi: 10.1038/s41591-019-0498-z PMC6688650 31270504

[142] Guo H Chou WC Lai Y Liang K Tam JW Brickey WJ. Multi-Omics Analyses of Radiation Survivors Identify Radioprotective Microbes and Metabolites. Science (2020) 370(6516):eaay9097. doi: 10.1126/science.aay9097 33122357 PMC7898465

[143] Burdelya LG Krivokrysenko VI Tallant TC Strom E Gleiberman AS Gupta D. An Agonist of Toll-Like Receptor 5 has Radioprotective Activity in Mouse and Primate Models. Science (2008) 320:226–30. doi: 10.1126/science.1154986 PMC4322935 18403709

[144] van Lier YF Davids M Haverkate NJE de Groot PF Donker ML Meijer E. Donor Fecal Microbiota Transplantation Ameliorates Intestinal Graft-Versus-Host Disease in Allogeneic Hematopoietic Cell Transplant Recipients. Sci Transl Med (2020) 12(556):aaz8926. doi: 10.1126/scitranslmed.aaz8926 32801142

[145] Markey KA Schluter J Gomes ALC Littmann ER Pickard AJ Taylor BP. The Microbe-Derived Short-Chain Fatty Acids Butyrate and Propionate are Associated With Protection From Chronic GVHD. Blood (2020) 136:130–6. doi: 10.1182/blood.2019003369 PMC7332893 32430495

[146] Yoshifuji K Inamoto K Kiridoshi Y Takeshita K Sasajima S Shiraishi Y. Prebiotics Protect Against Acute Graft-Versus-Host Disease and Preserve the Gut Microbiota in Stem Cell Transplantation. Blood Advances (2020) 4:4607–17. doi: 10.1182/bloodadvances.2020002604 PMC7556149 32991720

[147] Uribe-Herranz M Rafail S Beghi S Gil-de-Gomez L Verginadis I Bittinger K. Gut Microbiota Modulate Dendritic Cell Antigen Presentation and Radiotherapy-Induced Antitumor Immune Response. J Clin Invest (2020) 130:466–79. doi: 10.1172/JCI124332 PMC6934221 31815742

[148] Uribe-Herranz M Bittinger K Rafail S Guedan S Pierini S Tanes C. Gut Microbiota Modulates Adoptive Cell Therapy *via* CD8alpha Dendritic Cells and IL-12. JCI Insight (2018) 3(4):e94952. doi: 10.1172/jci.insight.94952 PMC5916241 29467322

[149] Suez J Zmora N Segal E Elinav E. The Pros, Cons, and Many Unknowns of Probiotics. Nat Med (2019) 25:716–29. doi: 10.1038/s41591-019-0439-x 31061539

[150] Yelin I Flett KB Merakou C Mehrotra P Stam J Snesrud E. Genomic and Epidemiological Evidence of Bacterial Transmission From Probiotic Capsule to Blood in ICU Patients. Nat Med (2019) 25:1728–32. doi: 10.1038/s41591-019-0626-9 PMC6980696 31700189

[151] Suez J Zmora N Zilberman-Schapira G Mor U Dori-Bachash M Bashiardes S. Post-Antibiotic Gut Mucosal Microbiome Reconstitution Is Impaired by Probiotics and Improved by Autologous FMT. Cell (2018) 174:1406–23 e16. doi: 10.1016/j.cell.2018.08.047 30193113

